# Baneful Effects of Saleratus and Cream of Tartar

**Published:** 1856-04

**Authors:** Samuel Baker


### BANEFUL EFFECTS OF SALERATUS AND CREAM OF TARTAR.

Two or three articles on this subject recently appeared in the *Portsmouth Journal*, N. H. We subjoin that part which more particularly relate to the effect of those articles on the teeth. We have several times made the experiment with these substances on teeth out of the mouth, and there can be no doubt but that they will very rapidly decompose them. We have several times referred to their destructive effects, and when we look at the severe tests to which the human teeth are daily subjected in civilized life, we are not astonished that they decay, but are rather astonished that so many save them at all. It appears to be only those most perfectly organized that can resist all the destructive agents to which they are subjected.

One of the most common questions asked of the dentist is, "why do people's teeth decay more now than they used to?" and it is often accompanied with the declaration, "that my father, mother or grand parents, used to have good teeth, and they say there were no dentists in their day;" and they might have added, there were neither of the above articles referred to much in use at that time.

It is now about fifty years since saleratus was introduced to the community. At that time there was but one dentist in Boston, and I may say in New England; he was sufficient for accidents, &c., and did a small business. There are probably at this time five hundred individuals in the business, and I should not be surprised (taking an average) that each does more business than he did. Now, why this great addition to the profession? Is it not by some great and general cause of the decay of the teeth? I am aware that a variety of answers might be made to this question, but too numerous to introduce here. I think if there was a soap introduced into the community (under the plea that it was more convenient) that should destroy the fingers, and the community still persist in using it, we should have another profession, called *fingerists*, that would rise up if loudly called for.

I may be answered that the population has increased. This is true; but not five hundred times. Again, I may be answered that people have more done to their teeth now than they used to. Very good; they have it done because they need it done,—but when they had "sound teeth" they did not want it. Again, it is said there are new inventions to preserve and supply teeth. Very well; new inventions do not come forth till the mother of invention calls for them. Children come into a dentist's office from two years old and upwards, with sets of decayed and often horribly painful and offensive teeth. Certainly age cannot have much to do with the alarming evil. *Something else is the cause.* On going on board emigrant vessels, I have generally observed the people have good teeth. After being here some time, they go to have their teeth extracted; and they often say that "my teeth did not decay before I came to this country." On asking them if they used saleratus or cream of tartar in their country, their answer has been invariably "no, never used it till I came here." I have three sculls that came from Paris, all of which have sound teeth, it would be difficult to obtain the like here as to teeth.

Apologists often say that we use but little saleratus, while one open hearted toothless woman observed, "I used to get it in a *box*, but I had to send so often I now get it in a *firkin*."

I do not think it generally known how much of the article is used in the community. To satisfy myself, I took the trouble to ask each of the grocers in Portsmouth how much saleratus and cream of tartar they sold in a year: and the amount of all was—*Saleratus*, fifty thousand one hundred and ninety-eight pounds; *Cream of Tartar*, fifteen thousand one hundred pounds. Thus, over twenty-five tons of the former and more than seven tons of the latter are probably used in Portsmouth and vicinity in a year! And I presume a like quantity in other places.

In ten years, 250 tons of one and 70 tons of the other pass over and around the teeth of the people of this city and adjoining towns! If its evil effects were known, I think it would be easy to convince the people that the amount was fully sufficient to carry on the business of destruction.

I subjected a handful of teeth to a strong and warm solution of saleratus for about fourteen days; the consequence was they became as brittle as burnt bones. The same time I subjected some to a solution of cream of tartar; the consequence was not the same, but equally if not more injurious. This also may be called an extreme case, but subjecting them to common water for *fourteen months* would have had but little or no effect on them.—The saleratus removes the gelatin, the cream of tartar removes the lime, the two principal ingredients of the teeth; and between the two evils the teeth stand a poor chance, and hence the result.

Some patients have observed that they have been recommended to put a lump of pearlash or saleratus in a hollow or decayed tooth, for the tooth-ache; and on asking them the result, "Why it for a time cured the pain, but in a few days the crown of the tooth crumbled away." It would appear that this one circumstance is sufficient to convince any one, that in the article there is a principle most deadly to the life of the teeth, when it is well known that the crown or enamel of a tooth is by far the hardest of any bone in the body, for after the other bones have returned to dust, this part has been found apparently sound.

Let the importance of the subject be the apology for offering a few more words; as they may be profitable to your readers. You will well remember our late venerable citizen, John McClintock, hale and hearty at over ninety.—I asked him if he, in his family, used saleratus or cream of tartar? His reply was, "No! THEY ARE BOTH POISON." All the physicians of the place whom I have conversed with, and among them the late Dr. Cheever, have agreed in saying that it was "bad stuff."—One gentleman in town having had a regular medical education, informed me that at the time he attended the lectures, the professors and students went into a course of experiments to inform themselves, and the result was that they were fully convinced of its hurtful and evil tendency. And when it has such apparently bad effect on the teeth, is it not reasonable to conclude that it has some effect on the other bones, and other parts of the system, and more particularly on the lining, or inner coating of the stomach, and in bringing on the somewhat modern disease dyspepsia; and more generally may it not affect the blood and heart?

One of the dealers observed, when he commenced business he used to buy some in a firkin, but now he bought it by the ton. He further said he was aware of the evil effects of it, and in his family they used but *very* little.

It may be well to say here, that cream of tartar may be and is called an acid, saleratus and carbonate of soda are alkalis; both alkalis and acids in a great variety of shapes and mixtures are prepared, advertised and recommended for cleaning the teeth. That they will generally do it, and sometimes suddenly, is true; but let me tell those that use for the teeth either acids or alkalies under whatever name or preparation, that it is generally well known, if persisted in a length of time, the general consequence is the destruction of the teeth; therefore, under all circumstances that relate to the teeth, have nothing to do with those ingredients in any shape.

To stop the use of the articles, saleratus, cream of tartar, and carbonate of soda, now in so general use in the community, I think would be like trying to remove the White

Mountains—but I feel it my duty to do my part towards calling the attention of the public to what seems to be a dreadful evil, of which, in my situation, I cannot help being so often and painfully reminded.

Since writing the above I have been asked if saleratus undergoes a change when used for cooking?—Probably, to some extent, but that its virulence is in force—particularly in some cases where it is used in bread, I should think that any one who has the senses of seeing, tasting, or smelling, and judgment enough to use them, would know of themselves, without any argument to prove it, or statement of the fact.

SAMUEL BAKER.

